# The Monthly Cycling of Food Insecurity in Latinas at Risk for Diabetes: Methods, Retention, and Sample Characteristics for a Microlongitudinal Design

**DOI:** 10.2196/66970

**Published:** 2025-03-28

**Authors:** Angela Bermúdez-Millán, Rafael Pérez-Escamilla, Sofia Segura-Pérez, James Grady, Richard S Feinn VI, Hanako Agresta, Dean Kim, Julie Ann Wagner

**Affiliations:** 1 Department of Public Health Sciences, School of Medicine, UConn Health, 263 Farmington Avenue, Farmington, CT, 06030-6325, United States, 1 8606797809, 1 8606791581; 2 Department of Social and Behavioral Sciences, Yale School of Public Health, New Haven, CT, United States; 3 Hispanic Health Council, Hartford, CT, United States; 4 Frank H. Netter School of Medicine, Quinnipiac University, Hamden, United States; 5 Division of Behavioral Sciences and Community Health, School of Dental Medicine, UConn Health, Farmington, CT, United States

**Keywords:** food insecurity, monthly cycling, type 2 diabetes risk, quantitative methods, Latinas, endocrinology, nutrition, nutrition assistance, micro-longitudinal design

## Abstract

**Background:**

Food insecurity (FI) is a risk factor for type 2 diabetes (T2D) that disproportionately affects Latinas. We conducted a microlongitudinal study to examine the relationship of monthly cycling of FI and diabetes risk factors.

**Objective:**

This study aimed to determine the quantitative methodology, recruitment and retention strategies, predictors of retention across time, and baseline sample demographics.

**Methods:**

Participants were adult Latinas living in Hartford, Connecticut who were recruited through a community agency, invited to participate if they were receiving Supplementary Nutrition Assistance Program (SNAP) benefits, screened positive for FI using the 2-item Hunger Vital Sign Screener, and had elevated risk factors for T2D using the American Diabetes Association risk factor test. Using a microlongitudinal design, we collected data twice per month for 3 months (week 2, which is a period of food budget adequacy; and week 4, which is a period of food budget inadequacy) to determine if the monthly cycling of FI was associated with near-term diabetes risk (fasting glucose, fructosamine, and glycosylated albumin) and long-term risk (BMI, waist circumference, and glycated hemoglobin) markers. We determined whether household food inventory, psychological distress, and binge eating mediated associations. We examined Health Action Process Approach model constructs. To assess the relationship between monthly cycling of FI with diabetes risk markers, we used repeated measures general linear mixed models. To assess the role of mediators, we performed a causal pathway analysis.

**Results:**

Participant enrollment was from April 1, 2021 to February 21, 2023. A total of 87 participants completed 420 assessments or a mean of 4.83 (SD 2.02) assessments. About half (47/87, 54%) of the sample self-identified as Puerto Rican, mean age was 35.1 (SD 5.8) years, with 17.1 (SD 11.6) years in the mainland United States. Just under half (41/87, 47.1%) spoke Spanish only, 69% (60/87) had no formal schooling, and 31% (27/87) had less than eighth grade education. Modal household size was 4 including 2 children; 44.8% (39/87) were not living with a partner. About half (47/87, 54%) were unemployed, 63.2% (55/87) reported a monthly income <US $1000, and 63.2% (55/87) used food pantries. In total, 61 participants (70.1%) completed all 6 assessments. On Pearson correlation analysis, having internet at home and having a tablet at home were associated with a higher number of completed assessments.

**Conclusions:**

This study demonstrated how FI cycles over the month and whether and to what degree the cycling itself is related to the risk for T2D development, as well as the evidence for some putative mechanisms of this association that can serve as future intervention targets including SNAP disbursement schedules.

## Introduction

Latinas have nearly double the rates of type 2 diabetes (T2D; 11.3%) compared with non-Hispanic white women (6.1%) [1]. This health inequity is not fully understood. Prediabetes, a condition in which a person’s blood glucose levels are higher than normal but do not reach the cutoff for diabetes, is a significant risk factor for developing T2D. In the United States, more than one-third of Latinas meet at least 1 American Diabetes Association criterion of prediabetes, and those younger than 45 years merit immediate attention for the prevention of advancing to full diabetes [2].

A risk factor for T2D that disproportionately affects Latinas is food insecurity (FI). A novel risk factor is the monthly cycling of food insecurity, an issue that has been notably understudied. FI is the limited or uncertain availability of nutritionally adequate and safe foods or the ability to acquire acceptable foods in socially acceptable ways [3,4]. In 2023, 13.5% (18.0 million) of US households were food insecure at least some time during the year [5]. Rates of FI were higher than the national average for households with children and particularly households with children headed by single adults, Hispanic-headed households, and households in principal cities.

FI has been associated with overweight or obesity, cardiometabolic disease, and T2D [6-8]. FI may influence diabetes risk in several ways. First, it may affect diet by promoting dependence on inexpensive, highly palatable foods that are energy dense, which are associated with weight gain and promote the development of chronic conditions [9]. Second, women in food insecure households may have elevated mental distress as a result of their central role in food acquisition and preparation for their families. The rates of depression and anxiety are higher among food insecure mothers, and among Latinos living in poverty [10]. Third, FI may affect emotional eating [11,12], the tendency to eat or overeat in response to negative emotions. Emotional eating has repeatedly been linked to overweight and obesity due to cycles of restriction and binge eating [12], resulting in fluctuations of meal size and frequency that contribute to weight gain.

A useful theoretical framework for describing the sociocognitive processes involved in health behaviors, such as fruit and vegetable intake, is the Health Action Process Approach (HAPA) model [13]. HAPA constructs include risk awareness, outcome expectancy, self-efficacy, intention, action planning, and coping planning. Understanding these constructs among Latinas in relation to FI may inform behavioral approaches for this population.

Most studies of FI have used cross-sectional or multiwave longitudinal designs, referencing the past 30 days, 3, 6, or 12 months, and have not captured the weekly ebb and flow of access to food, which may be crucial for understanding the role of FI as a diabetes risk factor, and for designing interventions. Yet, it has been hypothesized that among households that receive Supplemental Nutrition Assistance Program (SNAP) benefits, FI is not stable but cycles over the course of the month [14]. SNAP benefits are distributed in the beginning of the month and may not be sufficient to attenuate or prevent FI by the end of the month [15]. A small number of studies have shown that SNAP benefits lasting less than a month is a predictor of poor dietary quality and health outcomes [16] but this has not been studied in our population of interest. Our hypothesis is that FI cycling over the course of a month is detrimental to women’s health and is a risk factor for T2D.

Descriptive and formative research is crucial to address this potentially serious public health problem. Therefore, this innovative study aims to document FI occurrence over the month and test whether FI is associated with increases in diabetes risk markers in Latinas. Using a microlongitudinal design to capture the disbursement cycle of SNAP benefits, we collected data twice per month, the second week of the month which we expected to be a period of food budget adequacy and the fourth week of the month, which we expected to be a period of food budget inadequacy. We repeated this schedule for 3 months.

The first aim of the study was to determine if the monthly cycling of FI is associated with diabetes risk biomarkers. We hypothesized that compared with week 2 (food secure: period of food budget adequacy), week 4 (food insecure: period of food budget inadequacy) will be characterized by higher near-term diabetes risk biomarkers, that is, fasting glucose, fructosamine, and glycosylated albumin. We also hypothesized that compared with participants with little change in FI, participants with greater weekly changes in FI will have higher long-term metabolic risk markers for T2D (BMI, waist circumference, and glycated hemoglobin, HbA1c).

**Figure 1.**
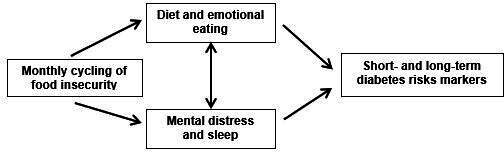
Conceptual framework for the Food Insecurity Cycling study (Hartford, Connecticut, enrollment 2021‐2023).

The second aim of the study was to describe the monthly cycling of household food inventory, eating behaviors, HAPA constructs, and distress. We hypothesized that compared with week 2 (food secure: period of food budget adequacy), week 4 (food insecure: period of food budget inadequacy) will be characterized by more eating disturbance (emotional eating and binge-eating), poorer diet quality (fat, sugar, fruit, and vegetables), and increased psychological distress (depression and anxiety symptoms; Figure 1). Changes in HAPA constructs over time are described. To our knowledge, this is the first microlongitudinal study to investigate FI cycling over time.

Longitudinal designs with multiple assessments can pose research burden on participants and are difficult to conduct in low-resourced populations. The aims of this paper are to report our quantitative methodology, to describe our recruitment and retention strategies, to examine predictors of retention across the 6 timepoints, and to report baseline demogrraphics for the sample.

## Methods

### Design

This study used a microlongitudinal design with 6 observations across 3 months.

### Participants

Eligibility criteria are shown in Textbox 1.

Textbox 1. **Inclusion criteria**

- Age 18‐44 years.
- Self-identify as Latina.
- Self-identify as a woman.
- Hartford area resident.
- Currently receiving Supplementary Nutrition Assistance Program (SNAP) benefits.
- Read and write in Spanish or English.
- Access to a smart phone to complete assessments.
- Screen positive for food insecurity per validated 2-item food insecurity screen [17,18] and at high risk for diabetes per standard criteria [19,20] (a score ≥4 indicates elevated risk for Hispanics). We studied adult Latinas of childbearing years because data suggest that they may prioritize nutrition for their children over their own nutrition [21].

**Exclusion criteria**

- Currently or recently pregnant or planning to become pregnant in the next 3 months (because of potential changes to nutrition benefits with pregnancy).
- Self-reported diabetes, kidney, or liver disease (because of nutritional requirements for management of those conditions).
- Positive screen for current substance abuse or dependence (to decrease attrition).
- Self-reported vasovagal syncope while undergoing phlebotomy (ie, history of feeling light-headed, dizziness, hot, sweaty, or fainting, to reduce medical urgencies associated with home phlebotomy).
- Living at a shelter (because women in a shelter do not have their own reliable food storage space).
- Those with previously undiagnosed diabetes who were identified at baseline as having unequivocal extant diabetes were excluded due to differences in cardiometabolic function and referred for treatment.

### Setting

As a US state, Connecticut is predominantly white, but its capital city of Hartford is largely Latino. Consistent with national data, Latinos in Connecticut have a significantly higher rate of diabetes than their white counterparts [22]. The study was led and coordinated by UConn Health and implemented in close partnership with the Hispanic Health Council (HHC) that used the study’s 2 bilingual and bicultural community health workers (CHWs). HHC is a community-based research, service, training, and advocacy organization headquartered in Hartford. This institutional partnership was built from a trusting relationship built over years of successful collaboration between UConn Health, Yale School of Public Health and HHC co-designing and implementing research studies using CHWs [23].

### Recruitment

A comprehensive staff training program was developed and delivered for HHC recruiters and data collectors, including a core manual to support fidelity to the assessment procedures and study protocol. CITI-trained, bilingual and bicultural recruiters distributed a recruitment flyer across HHC programs serving the study’s target population. The recruitment flyer briefly described the study and informed intersted individuals to call the recruiters. Programs included the Santa Marquez Special Supplemental Nutrition Program for Women, Infant, and Children Program (WIC) Center, which serves to safeguard the health of low-income women, infants, and children up to age 5 who are at nutritional risk. The recruitment flyer was also posted in the HHC social media platforms (ie, Hispanic Health Council Facebook, Instagram, and Twitter pages). HHC staff also distributed flyers at health fairs, Hartford mobile markets, community health agencies, local convenience stores, and local daycare centers and it was mentioned on Spanish-language radio. Recruitment also took place at local food pantries that serve the target community. Foodshare is the largest antihunger organization in the Greater Hartford area, with a network of food pantries, meal programs, and mobile foodshare sites.

### Screening and Enrollment

Eligibility screening was conducted by the CHW recruiters. Due to local rates of COVID-19 infection during the recruitment, most screening took place by phone, and some in a private room at the HHC. These sessions were scheduled through a virtual scheduling calendar accessible only to team personnel. The recruiters asked questions ascertaining the inclusion and exclusion criteria.

### Screening Measures

Demographic and clinical inclusion and exclusion criteria were self-reported. The 2-item Hunger Vital Sign FI screener was used to detect likely FI [17]. The 2 questions were “In the past 3 months, have you or other adults in your household worried whether your food would run out before you got money to buy more?” and “In the past 3 months, has the food you or other adults in your household bought just didn’t last and you didn’t have money to get more?” Response options were “yes,” or “no.” A response of “yes” to either question met study inclusion criteria for food insecurity [17].

The American Diabetes Association risk test was used to determine elevated risk for developing T2D. It includes seven self-report questions (total score of 0‐11) on age, gender, gestational diabetes mellitus, family history of diabetes, high blood pressure, physical activity, and obesity (based on BMI through a weight-height chart). The American Diabetes Association uses a cutoff of 5 or higher to indicate increased for diabetes. In this study, since all participants endorsed FI, which is itself a novel risk factor for diabetes, a score of ≥4 was required for inclusion [19,20].

The 4-item CAGE-AID (CAGE Adapted to Include Drugs) screener [24] was used to detect likely alcohol and substance abuse. A score of 2 is recommended as a positive screen for alcohol or substance abuse and indicates a potential problem that warrants further assessment by a health care professional; a score of 2 or higher excluded a recruit from participation.

### Assessment Procedures and Incentives

After obtaining consent to participate and HIPAA (Health Insurance Portability and Accountability Act) authorization, physical assessments were conducted in the participant’s preferred language (English or Spanish), at the HHC. Participants had their weight, height, waist, and hip circumferences measured and were asked to provide fasting blood samples. Assessments were performed early morning (ie, before 9 am) to obtain fasting blood samples (30 ml). Study phlebotomist from HHC took blood samples from an antecubital vein, using sterile technique. Samples were then promptly transported by trained personnel, compliant with HIPAA and biohazard regulations, in an ice chest to UConn Health Clinical Research Center for processing and analysis. There, blood samples were aliquoted. One aliquot went to UConn Health Hospital Clinical Laboratory for immediate assessment of glucose. The remaining aliquots went to the UConn Health Clinical Research Center where they were centrifuged and stored at −80°C until analyzed for all other assays described below.

Following the baseline assessment, participants provided data at 2 time points per month for 3 months, in order to capture the disbursement cycle of SNAP benefits. Participants received cash compensation for participation, US $15 at each timepoint for each blood draw and US $10 for surveys, totaling US $150.

### Retention Efforts

Because the temporal cycling of food insecurity over time is central to our research question, retention of this difficult-to-reach population was of utmost importance. Several community-engaged efforts were made to retain participants including the following: (1) Socializing the participant to the research endeavor for the community and describing the importance of each data collection timepoint; (2) Maintaining updated contact information for participants and a designated secondary contact person; (3) Speaking in the participant’s preferred language and fostering good rapport between CHWs and participants; (4) responding fully, genuinely, and in a timely manner to any participant concerns; (5) phone and text reminders for study appointments; and, (6) offering financial compensation for time spent on study activities.

### Measures

Next, participants were scheduled for a baseline assessment. Due to COVID-19 social distancing mandates, the majority of the survey instruments were collected over the phone by trained bilingual interviewers who entered participant verbal responses into REDCap (Research Electronic Data Capture) [25] on large-screen electronic tablets. For all self-report surveys, response options referenced the preceding 7-days. Refer to Table 1 for data collection timeline.

**Table 1.** Measures and assessment schedule.

Predictor	Month 1^a^		Month 2^a^		Month 3^a^	
	Week 1	Week 4	Week 1	Week 4	Week 1	Week 4
Food security^b^ (past 7 days timeframe)	✓	✓	✓	✓	✓	✓
**Covariates**						
Demographics	✓	N/A^c^	N/A	N/A	N/A	N/A
SNAP^d^ benefits	✓	✓	✓	✓	✓	✓
**Mediators (past 7 days timeframe)**						
Nutrition^e^	✓	✓	✓	✓	✓	✓
Distress (symptoms of anxiety and depression)	✓	✓	✓	✓	✓	✓
Sleep quality	✓	✓	✓	✓	✓	✓
**Outcomes (short-term)**						
Fasting blood glucose and insulin	✓	✓	✓	✓	✓	✓
Fructosamine	✓	✓	✓	✓	✓	✓
Glycosylated albumin	✓	✓	✓	✓	✓	✓
**Outcomes (long-term)**						
BMI	✓	N/A	N/A	N/A	N/A	✓
Waist and hip circumference	✓	N/A	N/A	N/A	N/A	✓
HbA1c	✓	N/A	N/A	N/A	N/A	✓

a SNAP benefits are made available on the electronic benefits transfer card from the 1st to the 3rd of every month, based on the first letter of the client’s last name. Data for week 1 were collected the week after food purchasing, during food abundancy; Week 4 data were collected before the next scheduled SNAP benefit disbursement, during food scarcity.

b 15-item US Household Food Security Survey Module.

c N/A: not applicable.

d SNAP: Supplementary Nutrition Assistance Program.

e Nutrition measures included food frequency questionnaire, household food inventory, HAPA model constructs, emotional eating, and binge eating.

#### Participant Characteristics at Baseline

Demographics included age (18‐24 years), language (English only, English and Spanish, or Spanish only), education (no formal schooling, eighth grade or less, some high school, high school graduate or GED [General Educational Development high school equivalency examination], some college, associates degree, or finished 4 years of college), employment (working full time or part time, unemployed, student, or homemaker), relationship status (living with a spouse or partner or not living with a spouse or partner), household composition (mother, father, mother-in-law, father-in-law, boyfriend/ or spouse, own children, sisters/, brothers, or others), and years living in the United States (free response). Financial strain was measured on a 5-point Likert scale [23] from 1= “We have enough and we can save” to 5 = “We don’t have enough and we have great difficulties.” Digital connectivity was measured with questions regarding whether the participant had internet and access to a computer and a tablet in the home, with response options yes or no.

#### Food Insecurity

Household food insecurity was assessed using a 15-item adapted and validated version of the original 18-item US Household Food Security Survey Module for pregnant Latinas [26,27]. The module captures the experience of anxiety regarding household food supplies, inadequate food quality and reduced food intake. This measure is validated in English and Spanish.

#### Nutrition Assistance

Participants reported how much in food stamps (SNAP) benefits they received per month, whether benefits were received once or twice per month, how many weeks the benefits last, whether and how much others in the household receive SNAP, and whether and how much the participant received from WIC.

#### Food Frequency Questionnaire

Diet quality was measured with the Spanish and English versions of the Block Fat, Sugar, Fruitt, Vegetable Screener, a 55-item food frequency questionnaire shown to have good reliability and validity [28,29]. Participants were instructed how to access and enter data through a link sent to them through email. Visual aids were uploaded in REDCap as images to help participants with portion size estimations [30].

#### Emotional Eating

The Emotional Eating subscale of the Three Factor Eating Questionnaire R18-V2 [31,32] assesses emotional eating. This subscale was validated in English and Spanish among Latinos of various Latino heritages [33]. The Spanish-language version [34] of the Questionnaire on Eating and Weight Patterns‐5 is a screening instrument for binge eating. It assesses eating‐disorder–behaviors and attitudes that align with DSM‐5 diagnostic criteria [35].

#### Psychological Distress

The validated Spanish version 8-item scale of the Patient-Reported Outcomes Measurement Information System emotional anxiety scale (Short Form 8a) assesses anxiety symptoms [36,37]. The validated Spanish version of the Personal Health Questionnaire Depression Scale (PHQ-8, ie, the PHQ-9 without the suicidality item) [38,39] assesses depressive symptoms. The suicidality item was omitted to avoid CHWs being required to respond to mental health urgencies in the field.

#### Sleep Quality

Sleep quality was measured with the Pittsburgh Sleep Quality Index (PSQI) [40]. The PSQI consists of 19 items that produce a global sleep quality score as well as individual items such as usual bedtime, wake-up time, actual hours slept, number of minutes to fall asleep, and nighttime awakenings. Global PSQI score was estimated for each participant as described previously, and as analyzed by our [41] team.

#### Health Action Process Approach Constructs

Questions were structured to assess motivational and volitional phases of the HAPA model as they pertain to eating behaviors. Specific items were adapted from previous studies of Latinas of childbearing age [42,43]. Four items tapped into risk awareness, for example, “If you continue with your current eating pattern, how likely is it that you will develop diabetes?” Response options were very unlikely, unlikely, likely, and very likely. Five items tapped into outcome expectancy, for example, “How true is it if you are able to improve and maintain healthier eating patterns, you will prevent diabetes?” Response options were not at all true, somewhat true, mostly true, and very true. Four items tapped into self-efficacy, for example, “I am certain or confident that I am able to improve my eating patterns, even if I have to make a detailed plan to have appropriate food available (on a food budget).” Response options were not at all true, somewhat true, mostly true, and very true. Three items tapped into intentions, for example, “During the next month, do you intend to increase the amount of vegetables you eat?” Response options were very unlikely, unlikely, likely, and very likely. Four items tapped into action planning, for example, “Do you have a plan of when you will go about eating more fruits and vegetables?” Response options were yes or no. Three items tapped into coping planning, for example, “Do you have a plan of how to incorporate more fruits and vegetables into your daily routine?” Response options were not at all, somewhat, or definitely.

#### Household Food Inventory

Participants completed a revised version of a home food inventory that has been validated in low-income Hispanic or Latinx households [44-46]. Participants completed the inventory themselves through REDCap [25]. Response options for each food were yes or no. Participants were instructed how to access and enter data through a link sent to them through email.

#### Anthropometrics

Weight was measured using a Seca Digital Scale and height was measured through a Seca Portable Stadiometer. Height was measured to the nearest 1 cm with the participant’s head positioned in the Frankfurt horizontal plane. Weight was measured to the nearest 0.1 kg. The average of 2 trials was used for both height and weight, unless measures were separated by a minimum of 1 cm or 0.5 kg, in which case, 2 more measures are taken. BMI (kg/m^2^) was calculated from height and weight. Waist circumference was measured twice at the umbilicus and hip circumference at the buttocks (nearest 0.5 cm).

#### Glucometrics

Fasting glucose reflects the body’s ability to regulate glucose in the absence of any glycemic challenge (ie, food). It is a strong predictor of incident T2D. Fasting glucose was analyzed at the UConn Health clinical laboratory. Fasting insulin was analyzed at the UConn Health Clinical Research Center with a kit from Siemens Healthcare Diagnostics (Los Angeles, California). It has a reference range median of 8.9 uIU/mL, sensitivity of 2 uIU/mL, and inter- and intrarun coefficients of variation (CVs) of 2.5 and 3.6, respectively. Fructosamine reflects the physiology of glucose in the extracellular space and has a short half-life, reflecting glycemia over 2‐4 weeks, ideal to address glycemia over intervals of several weeks. Fructosamine measures the fraction of total serum proteins that have undergone glycation. Assays were run at the UConn Health Clinical Research Center using a kit from ARUP laboratories (Salt Lake City, Utah) with an assay range 170‐285 μmol/L. Glycosylated albumin is formed by glycation of serum albumin. Several large, prospective studies have found that fructosamine and glycated albumin, a protein formed when glucose (sugar) binds to albumin, is a test that reflects short-term glycemia, predict conversion to T2D [47] with some studies demonstrating this effect even after controlling for baseline fasting glucose and HbA1c [48]. Glycated albumin was analyzed in serum at the UConn Health Clinical Research Center using a kit from MyBioSource Inc, San Diego, California. The assay has a range of 0.626‐20 mg/mL, with a sensitivity of 0.1 mg/mL. Intra- and interassay CVs are less than 15%.

#### Glycemia

HbA_1c_ was measured in the UConn clinical laboratory using high pressure liquid chromatography. In this laboratory, HbA_1c_ shows the following coefficients of variation for normal and high values: level 1 mean=5.51%, CV=3.3 based on n=320, and level 2 mean=9.01%, CV=3.2 based on n=304.

#### Insulin Resistance

Glucose was reported in mg/dL and insulin was reported in uiU/mL. Log transformed homeostatic model assessment of insulin resistance (logHOMA-IR, Homeostatic Model Assessment of Insulin Resistance) was calculated from fasting glucose and insulin values according to the standard formula: log(fasting glucose × fasting insulin)/405 [49].

### Sample Size and Power Analysis

Considering insulin resistance as our primary outcome, a previous study by our team provided data for a power analysis based on insulin resistance among persons at risk for T2D [50]. In that study, the SD for logHOMA-IR was approximately 0.3 and we expected to see a difference in logHOMA-IR of 0.4 between FI and food security, and a difference of 0.5. Our targeted sample size of n=100 would have 99% power to detect a main effect of FI, 96% power for mental distress and 87% power for the (FI × distress) interaction in a regression model using a 2-sided α of 0.05.

### Planned Data Analysis

Summary statistics will be used initially to present the results from the categorical and continuous variables. Skewness, kurtosis, and potential outliers will be examined and if needed transformations or categorical variables will be created. The internal consistency of self-report measures will be assessed by Cronbach α.

To assess the relationship between monthly cycling of FI with diabetes risk markers, we will use repeated measures general linear mixed models. With 3 months of data, collected at weeks 2 and 4, depending on the distribution of for each outcome variable, the most appropriate distribution and link function will be chosen. In separate analyses it will be possible to divide the sample by frequency of benefits (ie, once vs twice monthly benefits) and include a dummy variable to evaluate the effects of frequency of benefits in the statistical models. It will also be possible to divide the sample into food secure, intermittent and persistent FI based on longitudinal studies using this approach [51].

To assess the hypothesis that psychological distress, diet, and eating disturbance mediate the relationship between FI and diabetes risk, we will perform a causal pathway analysis. We are interested in examining whether there is a change in our mediators over time, and if so, whether this change significantly leads to a change in diabetes risk markers. For this we will conduct dynamic mediation using latent change (difference) score mediation analysis [52,53].

For food frequency measures, linear regression models will compare food security status on household food availability. Spearman rank-order correlations of total inventoried availability of fruit and vegetable servings with the food frequency questionnaire will be conducted. We plan to use “GENMOD” or “MIXED” procedures in SAS (SAS Institute), or similar, for regression models and MPLUS software (Muthén & Muthén), or similar, for the mediation causal pathway analysis.

### Ethical Considerations

If a recruit was not excluded, then recruiters obtained verbal consent, collected contact information, and scheduled the first visit at HHC. During this first visit, the recruiters obtained written informed consent. Participants were given a signed, dated copy of the informed consent form for their records and the recruiter kept the original, signed, dated copy. All verbal and written study materials, including recruitment materials and informed consent forms, were provided to the participants in their preferred language (English or Spanish). Self-report measures and their response options were pilot tested for readability, understanding, logic, flow, cultural relevance, and face validity by 5 Spanish speakers and 5 English speakers at the Hispanic Health Council. The final assessment battery was deemed appropriate by the research team. This protocol was approved by the UConn Health Institutional Review Board Study 21-045OS-1 and HHC agreed for UConn’s Health Institutional Review Board to be the institutional review board of record. All procedures were conducted according to the Declaration of Helsinki. Incentives were calculated to compensate participants for the time spent in study-related activities but were not coercive in a sample that screens positive for food insecurity (refer to Assessment Procedures and Incentives).

## Results

**Figure 2.**
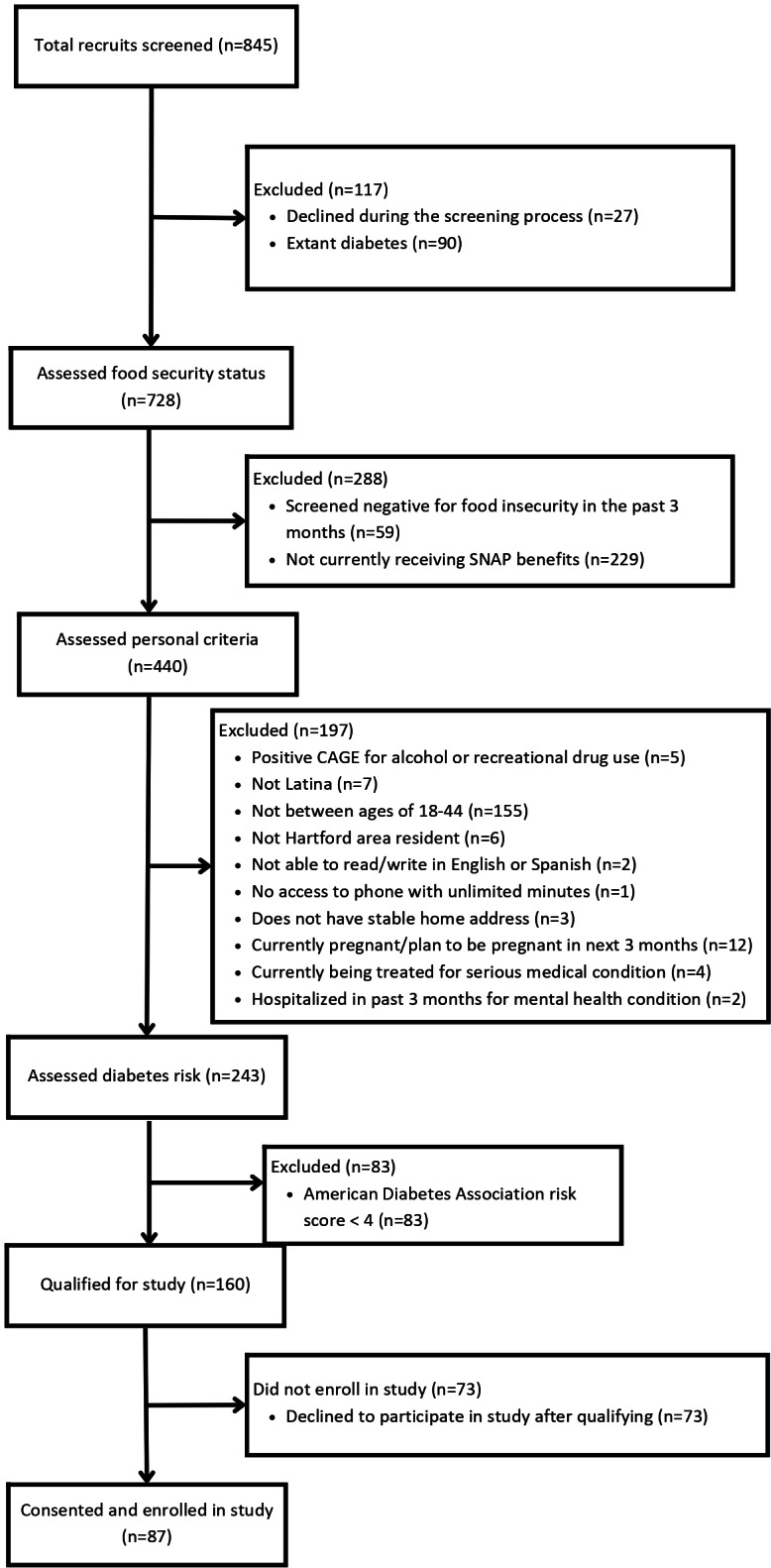
CONSORT diagram for the food insecurity cycling microlongitudinal study Hartford, Connecticut, enrollment 2021‐2023.

As can be seen in the diagram in Figure 2, 845 potential participants were screened. The majority (n=685) were not eligible, the most common exclusions being because they did not receive SNAP benefits (n=229), were outside of the age range (n=155), had extant diabetes (n=90), or did not have elevated risk for type 2 diabetes (n=83). Of the 160 participants who were eligible, 73 declined with the most common reasons for declining being lack of time, lack of transportation, and concerns about COVID-19 pandemic. A total of 89 participants were consented and completed baseline blood assays but 2 withdrew immediately afterwards; 87 of them went on to be enrolled.

As can be seen in Table 2, just over half (54%) of the sampled self-identified as Puerto Rican, mean age was 35.1 (5.8) years, with 17.1 (11.6) years in the mainland United States. Just under half (47.1%) spoke Spanish only, 69% had no formal schooling and 31% had less than eighth grade education. Modal household size was 4 including 2 children, and 44.8% were not living with a partner. About half (54%) were unemployed, 63.2% reported a monthly income <US $1000 and 63.2% used food pantries.

Overall, participants completed 420 assessments or mean=4.83 (SD=2.02) assessments. Of the 87, 70.1% (n=61) completed all 6 assessments. Results of Spearman’s correlations show that higher number of assessments completed was associated with having internet in the home (r=0.27, p=.01) and there was a statistical trend for having a tablet in the home (r=-0.20, p=.07).

**Table 2.** Demographic characteristics (n=87).

Characteristics	Values	
**Ethnicity n (%)**		
Puerto Rican	47 (54)	
Non-Puerto Rican	40 (46)	
Age, mean (SD), range (min-max)	35.1 (5.8) (18-44)	
**People Living in Home, n (%)**		
1	1 (1.1)	
2	7 (8)	
3	21 (24.1)	
4	24 (27.6)	
5	23 (26.4)	
6+	11 (12.6)	
**Children in home, n (%)**		
0	5 (5.7)	
1	16 (18.4)	
2	30 (34.5)	
3	24 (27.6)	
4+	12 (13.7)	
**Years in United States, mean (SD), range (min-max)**	17.1 (11.6) (0.3-43.3)	
**Language spoken, n (%)**		
English and Spanish	46 (52.9)	
Spanish only	41 (47.1)	
**Education, n (%)**		
No formal schooling	60 (69)	
Eighth grade or less	27 (31)	
**Relationship status, n (%)**		
Living with spouse or partner	48 (55.2)	
Not living with partner	39 (44.8)	
**Employment status, n (%)**		
Employed	39 (44.8)	
Unemployed	47 (54)	
No answer	1 (1.1)	
**Household income ($), n (%)**		
0-1000	55 (63.2)	
1000 or more	32 (36.8)	
**Financial strain, n (%)**		
Have enough and save	13 (14.9)	
Have just enough	48 (55.2)	
Not enough, with difficulties	25 (28.7)	
No answer	1 (1.1)	
**Financial assistance, n (%)**		
Cash	3 (3.4)	
Food Pantries	55 (63.2)	
Soup Kitchen	5 (5.7)	
Supplemental security income	15 (17.2)	
Title 19/Medicaid	54 (62.1)	
Section 8 housing	16 (18.4)	
Nutrition for women, infants, children and children (WIC)	35 (40.2)	
Other (housing and diapers)	4 (4.6)	
**Digital connectivity, n (%)**		
Internet in the home	82 (94.3)	
Tablet in the home	39 (44.8)	
Computer in the home	37 (42)	

## Discussion

### Principal Findings

The main findings reported here are that the study successfully recruited and retained a sample of the target population. There was excellent participation over time with 71% of the sample completing all 6 assessment timepoints. Participants were able to complete assessments using only their smart phones by cell phone service. Nonetheless, higher completion of assessments was associated with having internet and a tablet at home. This may reflect ease of connectivity (rather than access to connectivity) or a familiarity with technology that was advantageous. Other indicators of socioeconomic status (income and educational attainment) were not associated with number of assessments completed. As in other parts of the United States, digital inequity in Connecticut persists across race and ethnicity, income and education [54]. The surprisingly high rate of internet observed in this sample of Latinas who screened positive for food insecurity was likely due to assistance during COVID-19 pandemic for internet connection in the home. Future studies may achieve even higher rates of participation by providing tablets or internet service to participants, but the expense and ethical dilemmas of terminating internet service at the end of the study makes this approach potentially problematic.

This study was conducted during the COVID-19 pandemic. Social distancing requirements presented numerous challenges especially for recruitment, retention, and data collection. The study team remained flexible regarding format for assessments (telephone, Cisco WebEx, or in person when it was safe to do so) and strived to balance standardization with participant preference and evolving public health guidelines.

The innovative study protocol presented here will provide a comprehensive description of how FI is related to SNAP distribution, as well as, importantly, whether and to what degree FI cycling itself is related to risk for T2D. Furthermore, the study will provide early evidence for some putative mechanisms of this association, including contextual mechanisms such as household food availability, psychosocial mechanisms such as symptoms of depression and anxiety, and behavioral mechanisms such as distress-related binge eating. Each of these findings may suggest potential intervention targets.

### Novelty

Food insecurity is a known risk factor for onset of new T2D [55]. However, we postulate that food insecurity is not static; rather, it varies according to access to food which is largely driven by the reductions in the amount of money available from social assistance programs (including SNAP) over the course of the month in some instances with families finding themselves with hardly any economic resources left for food or other basic needs by the last week of the benefits monthly cycle. This cycling has been hypothesized in the literature [56] yet microlongitudinal data regarding FI cycling is not available, as far as we know (eg, [57]). Furthermore, we propose that this cycling is related not only to household food inventory, but also to distress and distress-related eating behaviors. Our group has documented that FI is associated cross-sectionally with higher symptoms of depression, anxiety, and stress hormones as well as lower sleep quality [41]. Yet it remains unknown whether FI and emotional distress covary over time.

Another novel contribution of this study is the procedure for the household food inventory. We taught participants to complete the inventory checklist themselves using REDCap [25]. Participants were instructed how to access and enter data through a link sent to them through email. Lessons learned regarding the feasibility of the method, and its fidelity, will be important to inform future studies using household food inventories with hard-to-reach populations, or studies when home visits by researchers are not possible due to pandemic, natural disaster, or other situation that disrupts home visits. We pilot tested items and omitted the items in the checklist that were not understood. However, were unable to conduct true fidelity checks to compare the participant responses with CHW responses, because the university protocols were not allowing home visits for research purposes. If found to be feasible and reliable, this method may also be more efficient for research teams and perhaps preferable for participants.

### Limitations

There are several limitations to this study. There is no experimental manipulation, so, whereas the longitudinal design can establish temporal precedence, no conclusions about causation can be drawn. This study was conducted during the COVID-19 pandemic. SNAP benefits since early 2020 have been affected by temporary pandemic-related benefit increases; an adjustment to the Thrifty Food Plan, upon which SNAP benefits are based, and higher-than-normal cost-of-living adjustments to reflect high food price inflation. Thus, findings may be influenced by changes to SNAP benefits during COVID-19 pandemic. COVID-19 pandemic also delayed and then paused the study, thus hindering recruitment. Data collection therefore took longer than anticipated and we fell short of our enrollment target by 13 participants. Future research with larger sample sizes should explore interactions between the exposure (FI) and mediators (psychological distress, diet, and eating disturbance) on outcomes (biomarkers of diabetes risk). Finally, the generalizability of our findings will need to be tested in other populations (eg, men, non-Latinos, or other parts of the country).

Limitations are balanced by the strengths of this study. The microlongitudinal design allows investigation of the temporal unfolding of covariance between FI and outcomes of interest over time. Home-based methods, particularly household food inventories, greatly improve contextual validity of assessments. The study affords the opportunity to confirm or assess the validity of certain measures in a Latina population, such as the Block Fat, Sugar, Fruit, and Vegetable Screener food frequency questionnaire. The population is high-risk and difficult-to-reach. Our procedures leverage the rigor and resources of a university medical center with the experience, knowledge, and reach of a community-based organization. Use of CHWs for recruitment, retention, and data collection improves trust and legitimacy in the target population. Multilevel bio-behavioral-psychosocial-contextual assessments are a particular strength. If our hypotheses are not refuted and this line of research goes on to establish causal pathways, interventions to disrupt the cycling of FI may prove beneficial for Latina health.

### Conclusions

Results of this study may have potential policy implications. In 2022, 375,400 Connecticut residents, or 10% of the state population, received SNAP benefits [58]. More than 50% of recipients were in households with children and more than 37% were working families. Findings of this study may have implications for SNAP policy, particularly amount and timing of SNAP benefit distribution. Qualitative findings show that SNAP benefits are often inadequate for participants to purchase nutrient-rich foods that are sufficient for 1 month of household intake, particularly for participants at the higher end of the SNAP income distribution. Two studies have found that participant responses to food security surveys depend upon when in the SNAP cycle they are queried, with higher reports of FI when SNAP benefits are most salient [56,59,60]. This suggests considerable variability in food security within the month.

Inadequate benefit levels may contribute to cyclical eating patterns, where participants increase food consumption when benefits are received and reduce food consumption when benefits are depleted [14]. It may also lead to the purchase of quite unhealthy ultraprocessed foods and sugar sweetened beverages that have a long shelf-life, rather than fresh fruits and vegetables that are more likely to spoil over the month. In another study, individuals were not receiving benefits at baseline and then received 3 months of benefits. At baseline, food expenditures were not cyclical and were not related to impulsivity. However, total food expenditures became cyclical after households started receiving benefits, with more impulsive shoppers demonstrating a more accentuated benefit cycle [15]. The current monthly distribution of benefits could be replaced with a biweekly distribution to address cyclical food security, food expenditures, and eating patterns. Such a disbursement would provide SNAP recipients with greater flexibility.

## Abbreviations

CAGE-AID: CAGE Adapted to Include Drugs
CHW: community health workers
CV: coefficient of variation
FI: Food insecurity
GED: General Educational Development high school equivalency exam
HAPA: Health Action Process Approach
HbA1c: glycated hemoglobin
HHC: Hispanic Health Council
HIPAA: Health Insurance Portability and Accountability Act
logHOMA-IR: Log transformed homeostatic model assessment of IR
PHQ: Personal Health Questionnaire
PSQI: Pittsburgh Sleep Quality Index
REDCap: Research Electronic Data Capture
SNAP: Supplementary Nutrition Assistance Program
T2D: type 2 diabetes
WIC program: Women, Infants, and Children program
